# Hyposmia and Cognitive Impairment in Gaucher Disease Patients and Carriers

**DOI:** 10.1002/mds.24945

**Published:** 2012-02-16

**Authors:** Alisdair McNeill, Raquel Duran, Christos Proukakis, Jose Bras, Derralyn Hughes, Atuhl Mehta, John Hardy, Nicholas W. Wood, Anthony H.V. Schapira

**Affiliations:** 1 Department of Clinical Neuroscience, University College London (UCL) Institute of Neurology, Royal Free Hospital, London, United Kingdom; 2 Department of Molecular Neuroscience, UCL Institute of Neurology, London, United Kingdom; 3 Lysosomal Storage Disorders Unit, Department of Haematology, Royal Free Hospital, London, United Kingdom

**Keywords:** Parkinson’s disease, genetics, olfactory dysfunction, cognitive dysfunction

## Abstract

The objective of this study was to assess a cohort of Gaucher disease patients and their heterozygous carrier relatives for potential clinical signs of early neurodegeneration. Gaucher disease patients (n = 30), heterozygous glucocerebrosidase mutation carriers (n = 30), and mutation-negative controls matched by age, sex, and ethnicity (n = 30) were recruited. Assessment was done for olfactory function (University of Pennsylvania Smell Identification Test), cognitive function (Mini-Mental State Examination, Montreal Cognitive Assessment), rapid eye movement sleep disorder, autonomic symptoms, and parkinsonian motor signs (Unified Parkinson’s Disease Rating Scale part III, Purdue pegboard). Olfactory function scores were significantly lower in Gaucher disease patients (*P* = .010) and heterozygous carriers (*P* < .001) than in controls. Cognitive assessment scores were significantly lower in Gaucher disease patients (*P* = .002) and carriers (*P* = .002) than in controls. Unified Parkinson’s Disease Rating Scale motor subscale scores were significantly higher in Gaucher disease patients (*P* < .001) and heterozygotes (*P* = .0010) than in controls. There was no difference in scores for symptoms of rapid eye movement sleep disorder or autonomic dysfunction. Impairment of olfaction, cognition, and parkinsonian motor signs occurs more frequently in Gaucher disease patients and carriers than in controls, which may indicate the early stages of neurodegeneration.

Gaucher disease (GD), the most common lysosomal storage disorder, is caused by recessive mutations in the glucocerebrosidase gene (*GBA*), which reduce activity of the enzyme beta-glucocerebrosidase (GCase).^1^ GD can be classified as non-neuronopathic (Type I) or neuronopathic (Type II and Type III). Recent studies have demonstrated that both Type I GD,^2,3^ and its heterozygous carrier state, predispose to Parkinson’s disease (PD) with Lewy body (LB) pathology,^4^ and heterozygous mutations have been found in 3.5% to 28% of postmortem cases of LB dementia (LBD).^5^ Bultron et al.^2^ found the age-adjusted lifetime risk of PD to be 21-fold greater in GD patients compared to controls, while Rosenbloom et al.^3^ demonstrated that GD patients at age 80 years have a 9% to 12% chance of developing PD. That heterozygous GBA mutations predispose to PD was confirmed by a multicenter analysis demonstrating that GBA mutations occur 5 times more frequently in patients with sporadic PD than in controls.^4^ The phenotype of PD associated with *GBA* mutations resembles sporadic PD.^6-8^

The motor features of PD develop when more than 50% of dopaminergic neurons in the substantia nigra pars compacta have degenerated.^9^ The motor syndrome of PD follows a prodromal period lasting up to 20 years. This period may feature non-motor symptoms such as hyposmia, autonomic symptoms (constipation, erectile dysfunction, syncope, urinary dysfunction), rapid eye movement (REM) sleep behavior disorder (RBD), and subtle cognitive impairment.^10,11^ Here we applied clinical screening tools for these symptoms, which have been identified as clinical markers of increased risk of developing a neurodegenerative disorder, to a group of individuals with GD and heterozygous mutation carriers to identify a cohort with clinical markers of potential early neurodegeneration.

## Patients and Methods

### Participants and *GBA* Sequencing

Type I GD patients, with no prior diagnosis of PD or dementia, were recruited from the Lysosomal Disorders Unit at the Royal Free Hospital, London. These individuals were homozygous, or compound heterozygous, for *GBA* mutations (Table 1). A family history was taken from each proband to identify relatives who could carry heterozygous *GBA* mutations. These individuals were recruited with the proband’s permission. Carriers were also recruited from the UK Gaucher Disease Association. Controls, matched to GD patients and carriers for age, sex, and ethnicity, were identified from relatives of GD probands. Controls had no neurological disease or systemic disease that could impair motor function. *GBA* mutation status in all participants was confirmed by sequencing exons 1 to 11 of the *GBA* gene, using a published protocol^6^ with polymerase chain reaction (PCR) primers designed exclusively for regions of the *GBA* gene not found in the pseudogene. After amplification by PCR the product was run on a 1% agarose gel with ethidium bromide and size-checked to ensure it was not the pseudogene. Sanger sequencing was performed for each exon and flanking intronic sequences using the Dye Terminator Sequencing Kit (Applied Bio-systems) on an ABI 3700xl genetic analyzer. This study had ethical approval from the North West London Research Ethics Committee. Written informed consent was taken from all participants.

### Clinical Evaluation

All procedures were performed identically in GD patients, carriers, and controls. Participants were assessed by a movement disorders–trained physician (A.M.). Participants were neurologically evaluated using the Unified Parkinson’s Disease Rating Scale activities of daily living and motor subscale (UPDRS parts II and III).^12^ Strict criteria were applied for the definition of PD: at least 2 of asymmetry, bradykinetic-rigid syndrome, and resting tremor, with excellent response to dopaminergic therapy (if treated).^9^

Odor identification was assessed with the 40-item University of Pennsylvania Smell Identification Test (UPSIT - Sensonics Inc, Haddon Heights, New Jersey, USA), which has been used in several published UK cohorts.^13^ Individuals with anatomical lesions of their upper airways, upper respiratory infections, or who were smokers were excluded. Cognitive function was assessed with the Mini-Mental State Examination (MMSE) and Montreal Cognitive Assessment (MoCA). The MoCA is more sensitive in detecting cognitive deficits in PD compared to the MMSE, with a score of <26 signifying mild cognitive impairment and <24 dementia.^14^ Features of RBD were screened for with the RBD Questionnaire.^15^ Autonomic dys-function was assessed with a subscale of the Unified Multiple System Atrophy Rating Scale (UMSARS), assessing orthostatic symptoms, impotence, urinary dysfunction, and constipation.^16^ The Purdue pegboard was used to quantify upper limb motor speed; the average of the number of pegs placed in 30 seconds using the right and left hands was scored.^17^

### Statistical Analysis

All analyses were performed using SPSS. Raw UPSIT scores, MMSE, MoCA, RBD, UMSARS, Purdue pegboard scores, and UPDRS II and III scores are not normally distributed. Differences between group medians were assessed with a Kruskal-Wallis test with the clinical marker as the dependent variable. Post hoc paired comparisons were performed with the Mann-Whitney U test using Bonferroni correction. Because 3 comparisons were made (control vs GD, control vs carrier, and carrier vs GD) significance at the 5% level was taken as a *P* value of .016. Student *t* test and the chi squared test were used to check differences in age, sex, and ethnicity between groups. Correlations between variables were assessed with a bivariate analysis using Spearman’s correlation. Significance was defined at the 5% level.

## Results

**FIG. 1:**
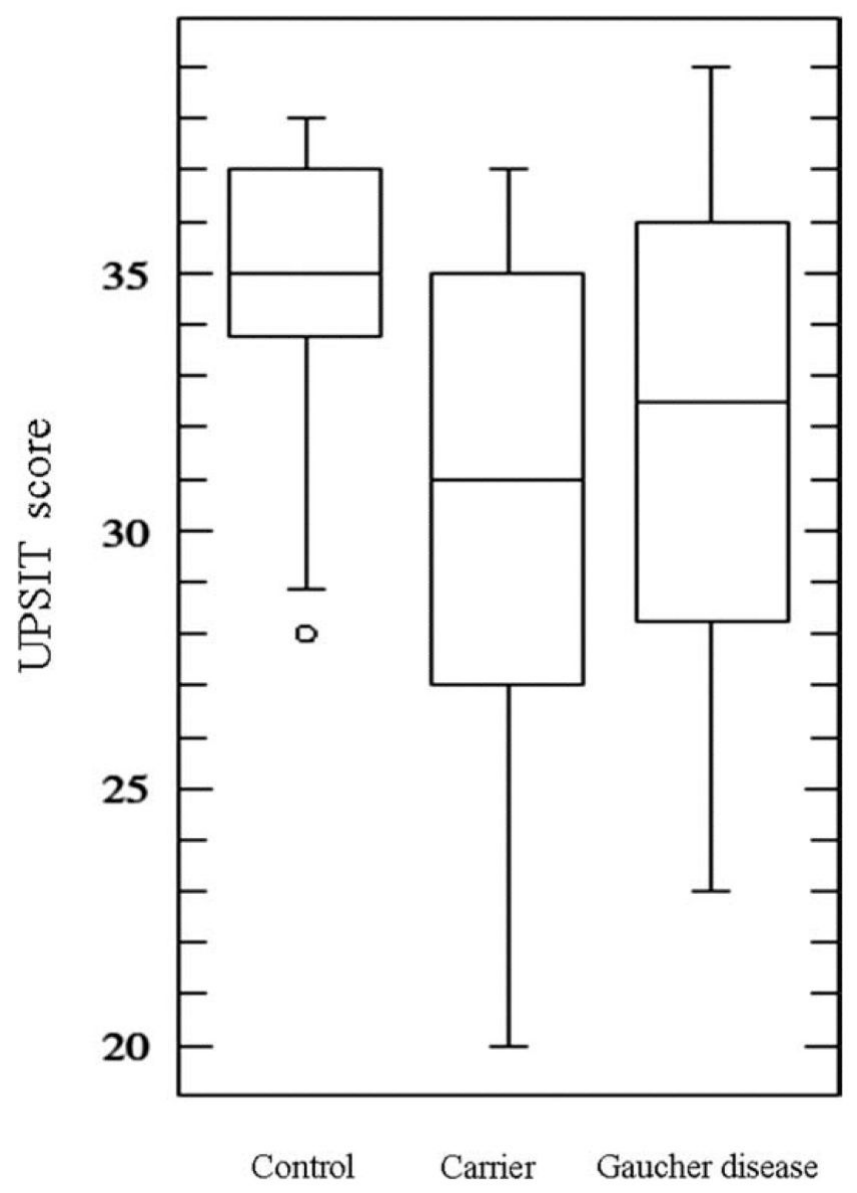
UPSIT scores for controls, carriers, and Gaucher disease patients. The median UPSIT score for controls was 35.5/40, which was significantly higher than the median UPSIT score for carriers (31/40; Mann-Whitney U test, *P* < .001) and Gaucher disease patients (32.5/40; Mann-Whitney U test, *P* = .01). The median UPSIT scores did not differ significantly between carriers and Gaucher disease patients. UPSIT, University of Pennsylvania Smell Identification Test.

**FIG. 2:**
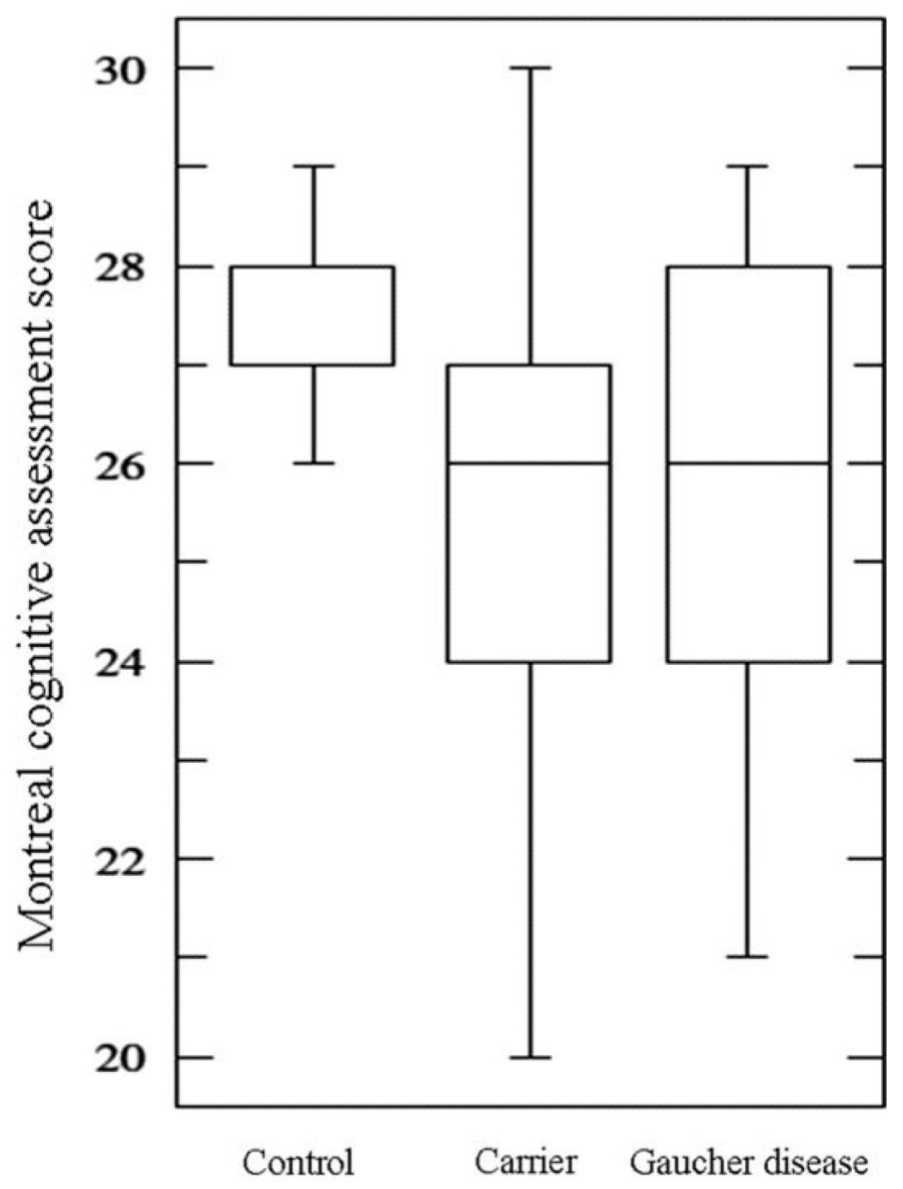
Montreal cognitive assessment scores for controls, carriers, and Gaucher disease. The median Montreal cognitive assessment score for controls (28/30) was significantly higher than that of carriers (26/30; Mann-Whitney U test, *P* = .002) or Gaucher disease patients (26/30; Mann-Whitney, *P* = .002). The median Montreal cognitive assessment scores did not differ between carriers and Gaucher disease patients.

**FIG. 3:**
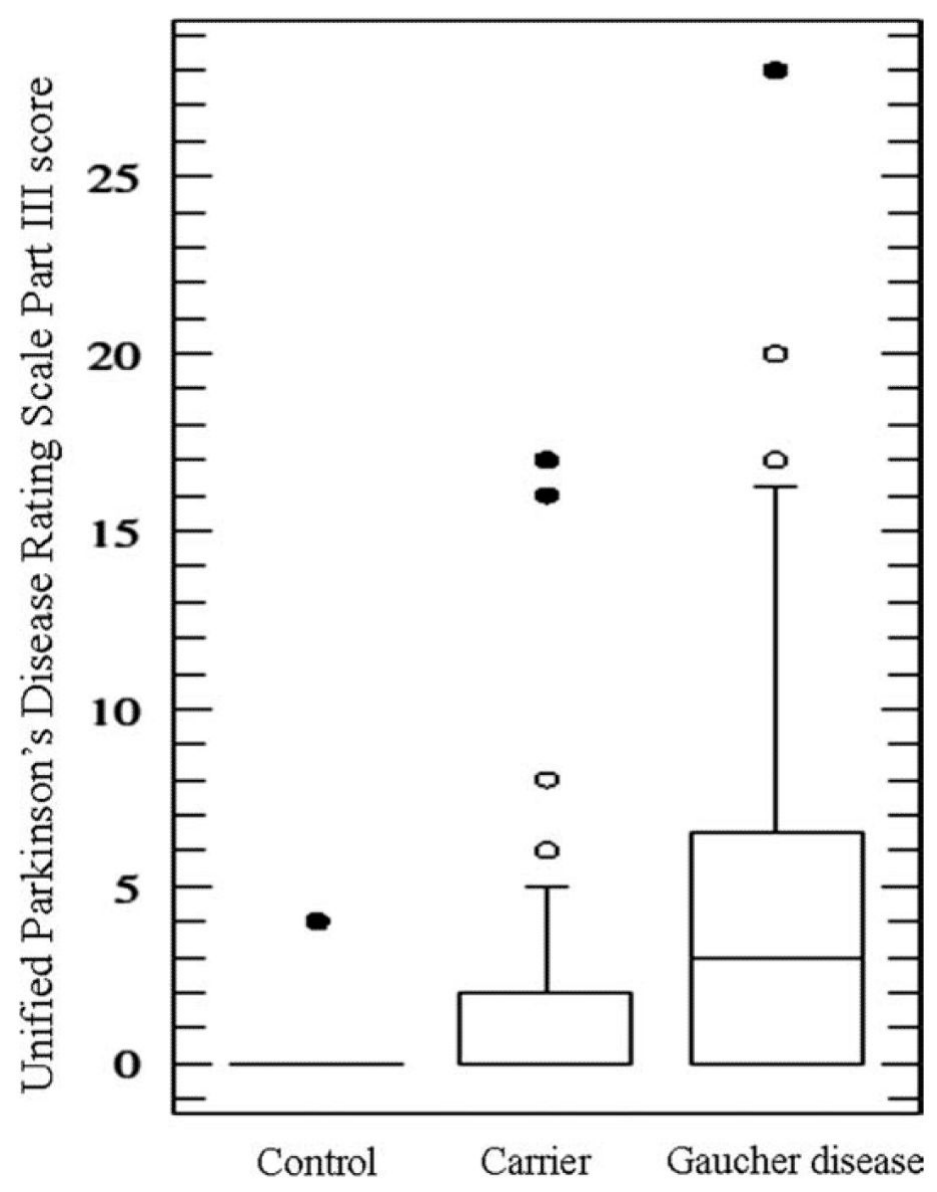
Unified Parkinson’s Disease Rating Scale Part III scores for controls, carriers, and Gaucher disease patients. The median Unified Parkinson’s Disease Rating Scale Part III score for controls was significantly lower than the median score for carriers (Mann-Whitney U test, *P* = .001) and Gaucher disease patients (Mann-Whiney U test, *P* < .001). The Unified Parkinson’s Disease Rating Scale Part III score did not differ significantly between carriers and Gaucher disease patients.

Thirty previously diagnosed Type I GD patients (male:female 14:16; mean age 61 years; range, 50–89 years) and 30 carriers (male:female 12:18; mean age 64 years; range, 50–80 years) were recruited. Thirty controls (male:female 13:17; mean age 63 years; range, 50–89 years) with no *GBA* mutations identified in exons 1 to 11 of the *GBA* gene were recruited, 7 of 30 controls were Ashkenazi Jewish; the remainder were white, UK-born individuals with no recorded Ashkenazi heritage. The age, sex, and ethnicities of the GD patients and carriers did not differ from each other or controls (Student *t* test and chi squared test, both *P* > .05). Of the GD patients 10 of 30 were of Ashkenazi Jewish descent and the remainder were white, UK-born individuals with no recorded Ashkenazi heritage (20/30). For GD patients, the majority (28/30) had at least 1 N370S allele, with the most common genotype being N370S/L444P (11/30) followed by N370S/N370S (9/30). No GD patients had features of Type III disease such as generalized seizures or progressive myoclonic epilepsy. Among carriers, 5 of 30 were Ashkenazi Jewish, with the remainder (25/30) white, UK-born individuals with no recorded Ashkenazi heritage. For carriers the most common genotype was N370S (14/30; 46%), followed by L444P (3/30; 10%) and recombinant alleles (2/30; 6%). Demographic, clinical, and molecular genetic variables for the cohort are presented in Table 1. Results of testing for clinical markers of neurodegeneration are summarized in Table 2 and Figures 1-3.

### Potential Neurodegenerative Markers in GD Patients Compared to Controls

Compared with controls, GD patients demonstrated an impairment of olfaction (Mann-Whitney, *P* = .01). Four GD patients were excluded from olfactory function testing (2 smokers, 2 coryzal illnesses). The MoCA demonstrated impaired cognition in GD patients versus controls (Mann-Whitney, *P* = .002); there was no difference in MMSE scores (Kruskal-Wallis, *P* = 0.95). Two GD patients declined cognitive function testing. Three GD patients had parkinsonian motor signs that did not meet diagnostic criteria for PD.^9^ GD05 had bilateral rigidity with activation maneuver, asymmetric bradykinesia of all limbs, and gait impairment. GD18 had left arm rest tremor and bilateral arm rigidity with activation maneuver. GD27 had flexed posture, bilateral rigidity, and postural and kinetic tremor of the upper limbs. In addition, UPDRS parts II (Mann-Whitney, *P* = .008) and III (Mann-Whitney, *P* < .001) scores were higher in GD patients than in controls. GD patients placed fewer pegs in the pegboard than controls (Mann-Whitney, *P* = .007). Two GD patients were excluded from the pegboard testing due to upper limb pain. There was no difference in the UMSARS score between GD and controls (Kruskal-Wallis, *P* = 0.11) or the RBD Questionnaire score between GD cases and controls (Kruskal-Wallis, *P* = .08). With GD05, GD18, and GD27 excluded from the analysis, GD patients’ olfactory test scores remained lower than controls (*P* = .025), their UPDRS part III scores were higher than controls (*P* < .001), and their MoCA scores were lower than controls (*P* = .001).

### Potential Neurodegenerative Markers in Heterozygous Carriers Compared to Controls

Compared with controls, heterozygous carriers demonstrated an impairment of olfaction (Mann-Whitney, *P* < .001). The MoCA demonstrated impaired cognition in carriers (Mann-Whitney, *P* = .002); there was no difference in MMSE scores (Kruskal-Wallis, *P* = 0.95). Two carriers had parkinsonian motor signs that did not meet diagnostic criteria for PD.^9^ C17 had bilateral rigidity, mask-like facies, and bradykinesia whereas C31 had masked facies, bilateral rigidity with activation maneuver, left arm kinetic tremor, and flexed posture. The UPDRS part II was not higher in carriers than controls (Mann-Whitney, *P* = .09), but the part III score was significantly higher in carriers than in controls (Mann-Whitney, *P* = .001). Carriers placed fewer pegs in the pegboard than controls (Mann-Whitney, *P* = .013). There was no difference in the UMSARS score between carriers and controls (Kruskal-Wallis, *P* = 0.95) nor was there a significant difference in the RBD Questionnaire score between carriers and controls (Kruskal-Wallis, *P* = .08). With C17 and C31 excluded, the olfactory test scores for carriers were lower than for controls (*P* < .001), the UPDRS part III scores were higher than for controls (*P* = .004), and the MoCA scores were lower than for controls (*P* = .001).

### Potential Neurodegenerative Markers in GD Patients Compared to Carriers

There was no significant differences in the median MoCA, MMSE, UPDRS parts II and III, UMSARS, or pegboard scores between GD patients and carriers (Mann-Whitney, all *P* > .05). There was no difference in the median UPSIT scores between GD patients and carriers (32.5 vs 31.0, Mann-Whitney, *P* > .05). Moreover, when assigned to categories of severity based upon age- and sex-adjusted centiles (www.sensonics.com) the profile of olfactory loss did not differ between GD patients and carriers. The proportion of GD patients and carriers with moderate hyposmia (UPSIT score 25–30/40, each 2/30, 6.6%), mild hyposmia (UPSIT score 25–30/40, GD 2/30 vs carrier 0/30, chi squared, *P* = 0.49), and severe hyposmia (UPSIT < 25/40, GD 2/30 [6.6%] vs carrier 5/30 [16.6%], chi squared, *P* = 0.14) were not significantly different. Two controls had mild hyposmia, no control had moderate or severe hyposmia.

### Correlations Between Olfactory, Cognitive, and Motor Function in GD Patients and Carriers

Several GD patients and carriers had more than 1 clinical marker of neurodegeneration. Statistically, the UPSIT score was correlated negatively with UPDRS part III (Spearman’s rho = −0.431, *P* = .02) in GD patients and carriers (Spearman’s rho = −0.394, *P* = .03); ie, lower olfactory function scores were associated with higher UPDRS part III scores. The UPSIT and MoCA scores did not correlate in GD patients (rho 0.083, *P* = .67) or carriers (rho = 0.176, *P* = .34).

## Discussion

Here we report neurological features relevant to increased risk of developing a neurodegenerative disorder such as PD in the largest cohort of GD patients and heterozygous *GBA* mutation carriers studied to date. We analyzed these and *GBA* mutation-negative controls for the presence of clinical markers of potential early neurodegeneration, demonstrating that hyposmia, cognitive dysfunction, and parkinsonian motor signs are prevalent among GD patients and heterozygous carriers. GD patients and carriers placed fewer pegs in the Purdue pegboard than controls; this can reflect loss of nigral dopaminergic neurons.^17^ These markers were heterogeneously distributed; a subset of individuals having more than 1 marker. This may reflect a mixed population, with only some individuals being in the early stages of a neurodegenerative disorder, and with those individuals having more than 1 marker most likely to be in the early stages of a neurodegenerative disorder. When outliers with the highest UPDRS part III scores were excluded, differences in olfaction, cognition, and UPDRS part III scores between GD patients and carriers compared to controls persisted, suggesting that our findings are not driven by a subgroup with overt motor parkinsonism.

The potential markers of neurodegeneration studied have diverse etiologies and a poor ability to predict development of PD.^10,11^ Only a minority of study participants with abnormal markers will be in the prodrome of a neurodegenerative disorder. Hyposmia has been described in GD patients with PD,^18^ but there are no reports of hyposmia in non-parkinsonian GD patients or as a side effect of GD treatment. The most common prodromal marker occurring in isolation was hyposmia (in 3/30 [10%] GD patients and 3/30 [10%] carriers), suggesting that hyposmia develops before cognitive dysfunction or motor abnormalities. We propose that hyposmia may be the most sensitive, but not a specific, marker for identifying GD patients and carriers at greatest risk of developing PD. Only a minority of individuals with idiopathic hyposmia go on to develop a neurodegenerative condition. In the Honolulu-Asia Aging Study, 10 of 549 hyposmic individuals developed PD,^19^ whereas Ponsen et al.^20^ found 10% of hyposmic first-degree relatives of PD patients developed PD themselves. Thus only a fraction of hyposmic GD patients and heterozygous carriers will have hyposmia due to a neurodegenerative prodrome. There are many non-neurological causes of hyposmia; we excluded smokers and those with symptomatic disease of their upper respiratory tracts, thus minimizing potential confounding variables.

Cognitive dysfunction has been reported in Type I GD, and substrate reduction therapy (SRT) may induce memory problems.^21^ However, only 2 GD patients were receiving SRT when assessed, and neither had cognitive impairment. A study of 20 PD patients carrying the N370S or L444P mutation found cognitive impairment by MoCA and other neuro-psychiatric features to be more common than in PD patients without *GBA* mutations.^22^ Thus heterozygous and biallelic *GBA* mutations are associated with cognitive dysfunction. It is unclear whether the cognitive dysfunction identified relates to a manifestation of Type I GD per se or the early phase of a neurodegenerative disorder such as PD.

It is recognized that the UPDRS part III differentiates poorly between subtle Parkinsonian signs and nonspecific motor features occurring as part of normal aging. Among study participants who scored 0 to 10 on the UPDRS part III there was a mixture of individuals with signs that were highly likely to reflect early motor features of PD (eg, rigidity with activation maneuver) and those whose motor features overlapped with normal physiology (eg, bilateral postural tremor) or might reflect bone disease in GD (eg, postural abnormalities). Despite this, there were 3 GD patients and 2 carriers with UPDRS part III scores >10 with motor phenotypes falling short of diagnostic criteria for PD (eg, rest tremor with asymmetric rigidity but no bradykinesia) but clearly distinct from normal. We do not propose that all subjects with elevated UPDRS part III scores will develop PD, but we hypothesize that those with clear motor abnormalities (eg, rest tremor) are more likely to develop neurodegeneration. Given that PD is associated with hyposmia and cognitive impairment, we believe study participants with combinations of hyposmia, cognitive dysfunction, and motor signs are most likely to be in the early stages of a neurodegenerative disorder.

We did not identify an increased frequency of symptoms of autonomic dysfunction or RBD in the GD patients and carriers. This does not undermine our contention that we identified early markers of neurodegeneration in this cohort. In the majority of cases urinary and erectile dysfunction develop after onset of motor PD.^10,11^ In 1 study of PD patients with autonomic dysfunction, orthostatic hypotension was only present in 60% as an early feature. Constipation is well-recognized as a marker of increased PD risk, but it has low specificity.^10^ Autonomic symptoms are often more associated with clinical PD than the prodrome, and their absence does not preclude increased risk of neurodegeneration. RBD correlates with risk of developing neurodegeneration.^10^ There is evidence the RBD may define an endophenotype of PD, with 25% to 50% of PD patients having clinical features of the disorder.^10,11^ Thus not all individuals in the prodrome of neurodegeneration will have RBD and its absence does not indicate low risk of neurodegeneration. We screened for a range of neurodegenerative markers and could not examine them in detail (eg, autonomic function testing). This may explain why we did not identify autonomic dysfunction/RBD: the screening tools do not identify all cases.^10,15^

Both biallelic and heterozygous *GBA* mutations predispose to PD and LBD.^2-5^ The olfactory deficits, cognitive dysfunction, and parkinsonian signs in our cohort may represent the prodrome of 1 of these disorders. Olfactory dysfunction is well-recognized in established and prodromal PD. In the Honolulu-Asia Aging Study, hyposmia (lowest quartile of odor identification scores) gave an odds ratio (OR) of 5.2 for the development of PD over a 4-year period and with idiopathic LB pathology at autopsy.^19^ Hyposmia also occurs in LBD, distinct from odor identification/naming difficulties associated with dementia.^23^ Olfactory dysfunction in PD and LBD is associated with LB deposition in the olfactory bulb, nucleus, and cortex.^24^ Several GD patients and carriers had mild cognitive impairment (MCI), defined by the MoCA (score of <26/30). MCI is common in newly diagnosed PD (18%-30%).^25^ A distinct MCI phenotype is proposed as a prodrome to LBD.^23^ Cortical LB pathology is associated with cognitive impairment in PD and LBD.^26^ GD patients and carriers have clinical signs associated with LB deposition or neuronal loss linked to future development of PD or LBD.

How *GBA* mutations predispose to PD and LBD remains unclear.^2,3^ In GD it is hypothesized that biallelic *GBA* mutations cause lysosomal dysfunction, impaired alpha-synuclein degradation, and impaired LB formation.^27^ Enzyme replacement therapy cannot penetrate the blood brain barrier, so this hypothesis remains valid in treated GD patients. Although heterozygotes have sufficient residual GCase activity to prevent systemic manifestations of GD it is possible that reduced GCase activity in these individuals impairs lysosomal function or results in substrate deposition that might contribute to PD pathogenesis. The demonstration of abnormal expression of lysosomal markers in PD brain in areas mapping to neuronal degeneration and LB deposition highlights the importance of this pathway.^28^ The most common GD allele found in sporadic PD is N370S.^4^ This mutation causes protein misfolding and has the highest residual GCase activity of any *GBA* mutation.^4^ This suggests protein misfolding and endoplasmic reticulum stress may contribute to PD risk in heterozygotes. In conclusion, our study provides evidence that GD patients and heterozygote *GBA* mutation carriers exhibit certain features consistent with the PD clinical prodrome. That both homozygotes and heterozygotes are indistinguishable in this respect is of significance, implying that severity of *GBA* enzyme deficiency is not the determining factor for PD risk in these individuals.

## Figures and Tables

**TABLE 1:** Demographic, clinical, and genetic characteristics of Gaucher disease patients

Case	Age, sex	Ethnicity	Age of onset (yr)	Genotype	Splenectomy	Treatment
GD01	56, F	Ashkenazi	24	N370S/N370S	No	ERT
GD02	66, M	Ashkenazi	47	N370S/N370S	Yes	ERT
GD03	69, F	Ashkenazi	16	N370S/L444P	Yes	ERT
GD04	56, F	White	16	N370S/84GG	Yes	ERT
GD05	76, M	Ashkenazi	10	N370S/N370S	No	ERT
GD06	50, M	White	48	L444P/RecNil	No	ERT
GD07	88, F	Ashkenazi	87	N370S/ins intron 6	No	Nil
GD08	88, F	Ashkenazi	73	N370S/N370S	No	ERT
GD09	50, M	White	5	R463C/RecNil	Yes	ERT
GD10	54, F	White	6	N370S/V447E	Yes	SRT
GD11	71, M	Ashkenazi	NA	N370S/N370S	No	ERT
GD12	56, F	White	30	N370S/L444P	No	ERT
GD13	50, F	White	51	N370S/G250V	Yes	ERT
GD14	61, F	White	5	N370S/N370S	Yes	ERT
GD15	48, M	White	27	N370S/L444P	Yes	ERT
GD16	51, F	White	38	N370S/?	No	Nil
GD17	53, M	White	45	N370S/84GG	No	Nil
GD18	81, M	Ashkenazi	5	N370S/L444P	No	ERT
GD19	84, F	Ashkenazi	80	N370S/N370S	No	ERT
GD20	52, M	White	50	N370S/L444P	No	ERT
GD21	53, F	White	38	N370S/L444P	No	ERT
GD22	58, M	White	40	N370S/N370S	No	ERT
GD23	56, F	White	NA	N370S/L444P	No	ERT
GD24	53, F	Ashkenazi	34	N370S/N370S	No	SRT
GD25	60, M	White	44	N370S/203InsC	No	ERT
GD26	57, M	White	30	N370S/P182T	No	ERT
GD27	67, M	White	28	N370S/L444P	No	ERT
GD28	50, F	White	30	N370S/L444P	No	ERT
GD29	56, F	White	32	N370S/L444P	No	ERT
GD30	58, M	White	27	N370S/L444P	No	ERT

ERT, enzyme replacement therapy; NA, not available; Nil, no treatment; SRT, substrate reduction therapy.

**TABLE 2:** Potential markers of neurodegeneration in GD patients and carriers

	Controls (n = 30)	Carriers (n = 30)	GD (n = 30)	Post hoc significance (<.05)^a,b^
Age (yr), mean (range), males:females	63 (50–89), M13:17F	64 (50–80), 12M:18F	61 (50–89), 14M:16F	No significant difference
Non-motor scores, median (interquartile range)				
Olfaction^a^	35.5 (33.75–37.0)	31.0 (25.0–35.0)	32.5 (26.75–36.0)	A>B, A>C
MMSE	29.0 (29.0–30.0)	29.0 (28.5–30.0)	29 (28.25–30.0)	No significant difference
MoCA^a^	28.0 (26.25–28.0)	26.0 (24.0–27.0)	26.0 (24.0–27.75)	A>B, A>C
UMSARS	0.0 (0.0–0.0)	0.0 (0.0–0.0)	0.0 (0.0–1.0)	No significant difference
RBD	0.0 (0.0–0.0)	0.0 (0.0–0.0)	0.0 (0.0–0.0)	No significant difference
Motor scores, median (interquartile range)				
UPDRS II	0.0, 0.0	0.28 ± 0.16	1.2 ± 0.66	C>A
UPDRS III^a^	0.0 (0.0–0.0)	0.0 (0.0–2.0)	3.0 (0.0–7.0)	B>A, C>A

All results are presented as median and interquartile range. Significance was taken at the 5% level for all variables.

a With Student *t* test or chi-squared test for age/sex, and Mann-Whitney U test with Bonferroni correction for markers of neurodegeneration.

b A = controls; B = carriers, and C = GD patients.

GD, Gaucher disease; MMSE, Mini-Mental State Examination; MoCA, Montreal Cognitive Assessment; UMSARS, Unified Multiple System Atrophy Rating Scale; RBD, REM sleep behavior disorder; REM, rapid eye movement; UPDRS, Unified Parkinson’s Disease Rating Scale.
